# Photons from NIR LEDs can delay flowering in short-day soybean and *Cannabis*: Implications for phytochrome activity

**DOI:** 10.1371/journal.pone.0255232

**Published:** 2021-07-27

**Authors:** Paul Kusuma, F. Mitchell Westmoreland, Shuyang Zhen, Bruce Bugbee

**Affiliations:** 1 Department of Plants Soils and Climate, Crop Physiology Laboratory, Utah State University, Logan, UT, United States of America; 2 Department of Horticultural Sciences, Texas A&M University, College Station, TX, United States of America; United Arab Emirates University, UNITED ARAB EMIRATES

## Abstract

Photons during the dark period delay flowering in short-day plants (SDP). Red photons applied at night convert phytochromes to the active far-red absorbing form (P_fr_), leading to inhibition of flowering. Far-red photons (greater than 700 nm) re-induce flowering when applied after a pulse of red photons during the dark period. However, far-red photons at sufficiently high intensity and duration delay flowering in sensitive species. Mechanistically, this response occurs because phytochrome-red (P_r_) absorbance is not zero beyond 700 nm. We applied nighttime photons from near infrared (NIR) LEDs (peak 850 nm) over a 12 h dark period. Flowering was delayed in *Glycine max* and *Cannabis sativa* (two photosensitive species) by 3 and 12 days, respectively, as the flux of photons from NIR LEDs was increased up to 83 and 116 μmol m^-2^ s^-1^. This suggests that long wavelength photons from NIR LEDs can activate phytochromes (convert P_r_ to P_fr_) and thus alter plant development.

## Introduction

**Fig 1:**
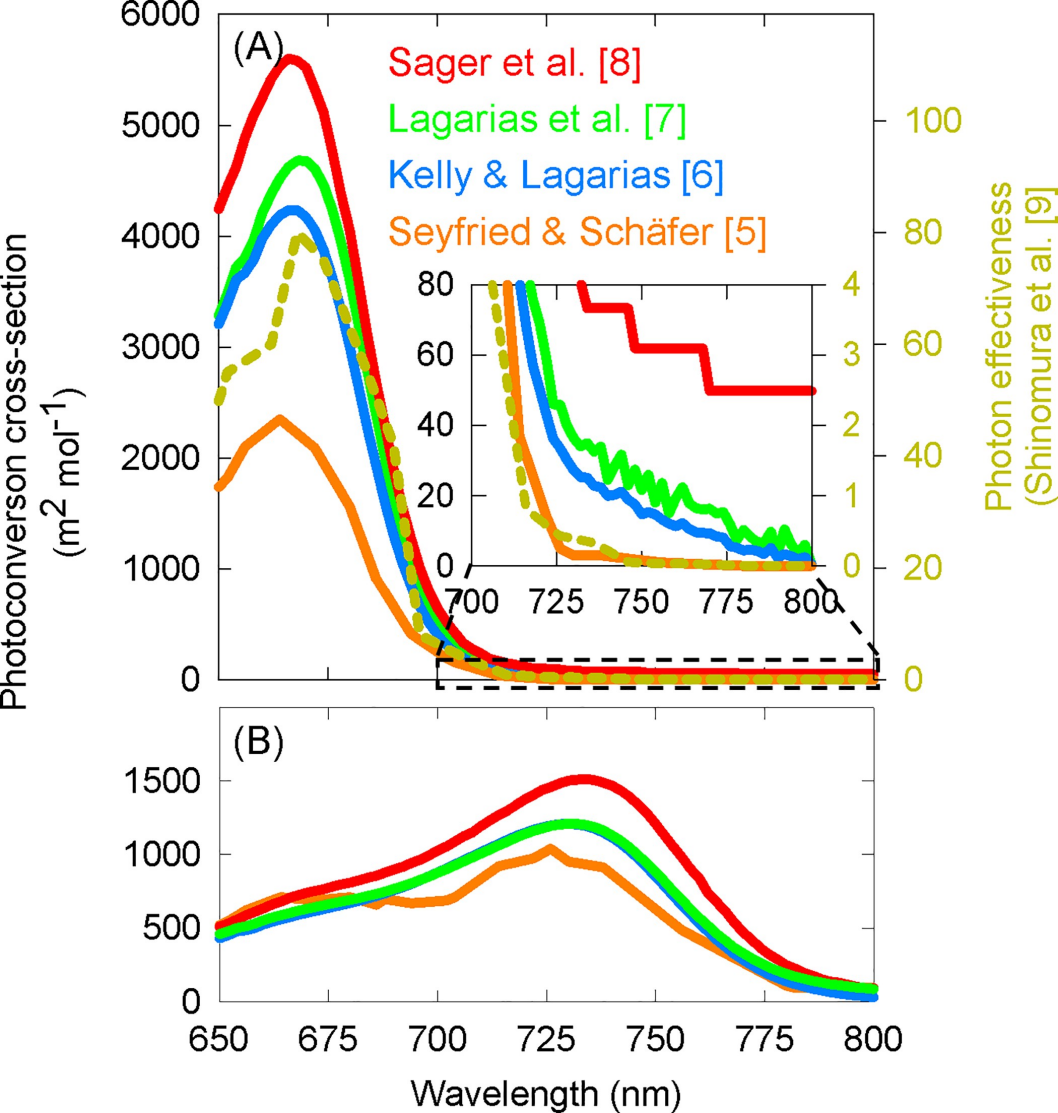
(A) Left axis, photoconversion cross-sections of P_r_ determined by Seyfried and Schäfer [5], Kelly and Lagarias [6], Lagarias et al. [7] and Sager et al. [8]. Photoconversion cross-sections are related to absorbance spectra. Right axis, action spectrum of seed germination (dashed line) [9]. Inset: P_r_ photoconversion cross-section between 700 to 800 nm. Note the inconsistency of P_r_ action above 750 nm determined by different research groups. (B) Photoconversion cross-sections of P_fr_.

Phytochromes are a class of plant photoreceptors that modulate development throughout the life cycle of a plant. They interconvert between two major forms upon photon absorption: the inactive form (P_r_), which is most sensitive to red photons, and the active form (P_fr_), which is most sensitive to far-red photons [1]. Although P_r_ and P_fr_ are named for the region that they are most sensitive to, both forms absorb across the entire biologically active range of radiation (300 to 800 nm). Historically, a metric called phytochrome photoequilibrium (PPE) has been used to predict phytochrome-mediated developmental responses [2]. PPE is an estimate of the fraction of active P_fr_ to the total phytochrome pool, and it is calculated from the spectral photon distribution (SPD) of the incident light and photoconversion cross-sections (which predict the likelihood of photon absorbance and subsequent phytochrome conversion) for P_r_ and P_fr_ at each wavelength [2–8]. Photoconversion cross-sections are closely related to absorption spectra and, when multiplied by the photon intensity at specific wavelengths, they provide an estimate of the rates of conversion between the two forms of phytochrome [3, 4]. Several studies have separately derived the photochemical parameters necessary to calculate these photoconversion cross-sections. Fig 1A shows four sets of P_r_ cross-section values (from 650 to 800 nm) that are derived from 1) Seyfried and Schäfer [5], 2) Kelly and Lagarias [6], 3) Lagarias et al. [7] and 4) Sager et al. [8]. Shinomura et al. [9] used a spectrograph (a device that uses prisms to provide narrow bandwidths of radiation) to determine the action spectrum of seed germination in *Arabidopsis thaliana* and found that it closely matched the absorbance spectrum of P_r_ (Fig 1A). Phytochrome absorbance spectra above 800 nm have not been rigorously determined, but Schäfer et al. [10] predicted the photoconversion cross-sections out to 1100 nm using action spectra responses for both the inhibition of mesocotyl elongation and the promotion of coleptile elongation. These data showed a sustained decrease in the relative photoconversion cross-section out to 1100 nm. This indicates that, although the ability of photons to activate P_r_ into P_fr_ decreases rapidly above 700 nm (and even 800 nm), responses ought to still occur beyond 700 nm with high enough photon intensities.

Photoconversions for the conversion of P_fr_ back to P_r_ are also available from many of these studies [5–8] (Fig 1B). These show a higher sensitivity than P_r_ cross-sections between 700 to 800 nm, but similar to P_r_, rigorously determined P_fr_ photoconversion cross-sections are not available for wavelengths above 800 nm.

**Fig 2:**
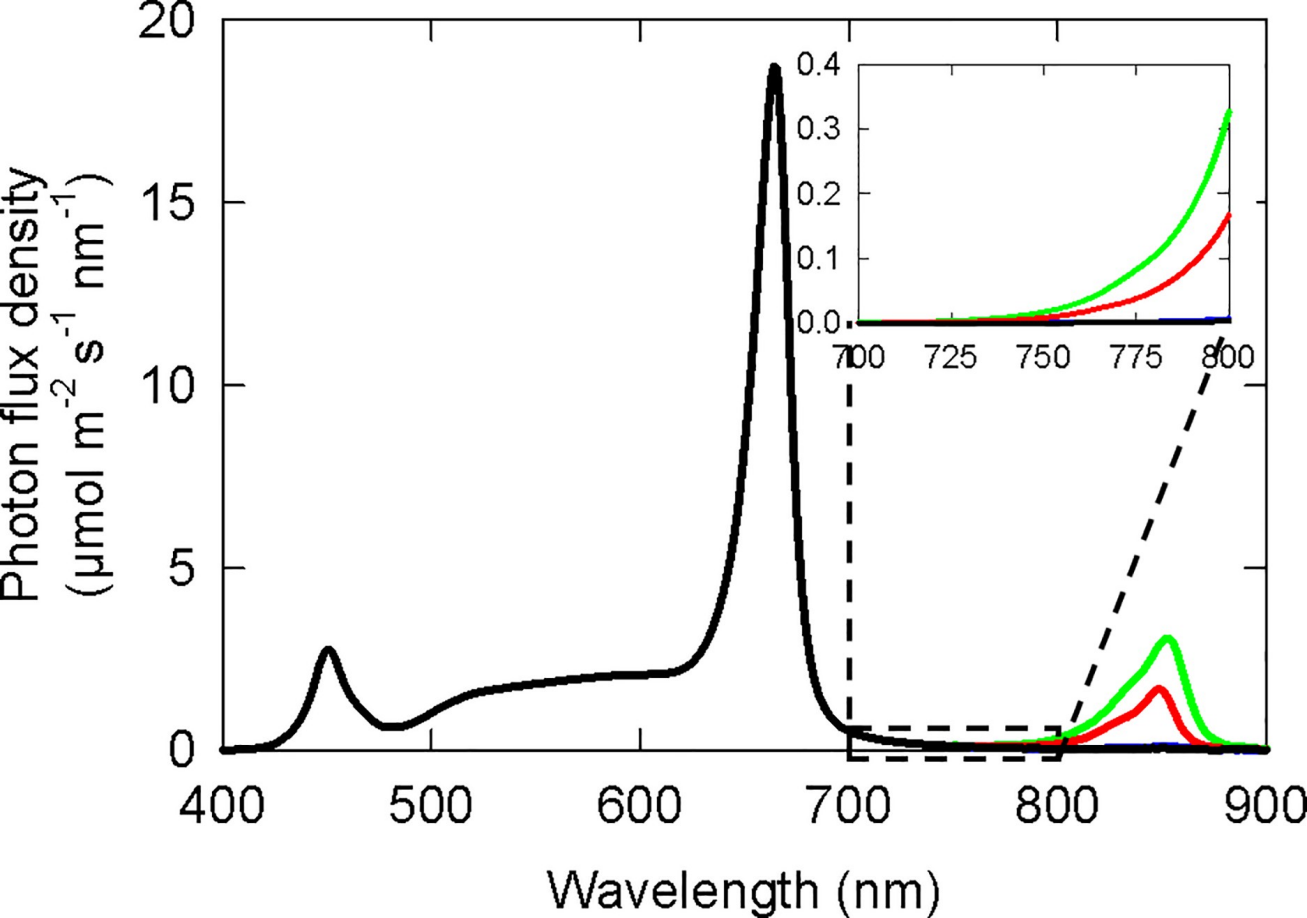
Spectral distribution from cannabis studies. The black line is the background spectral distribution used in all treatments including the control. The red and green lines show the two intensities of added photons from a near-infrared (NIR) LED (low NIR and high NIR in Table 2) across the three replicate studies. Inset: Spectral distribution between 700 and 800 nm of nighttime light pollution from NIR LEDs. Spectral distributions in the soybean study had the same shapes but with lower overall intensities.

A common NIR LED for night vision has a peak at about 850 with a full width at half maximum (FWHM) of about 35 nm and outputs photons down to about 700 nm (S1 Fig). This LED is used in security cameras for night vision in greenhouses and controlled environment agriculture. Because of their regular use in controlled plant growth environments, especially during the dark periods, it is valuable to investigate the potential role they may play in altering plant growth and development. The photons from this LED may affect plant growth and development either by 1) the activation of P_r_ to P_fr_ (Fig 1A), or 2) the inactivation of phytochrome from P_fr_ to P_r_ (Fig 1B). The activation of phytochromes (i.e. convert P_r_ to P_fr_) can be assessed by the inhibition of floral initiation in short-day plants (SDP), while the inactivation of phytochrome (i.e. convert P_fr_ to P_r_) can be assessed with stem elongation.

SDP undergo floral initiation when the period of un-interrupted darkness is longer than a critical length [11]. The application of photons for 4 hours or less during the dark period, called a night-break or night-interruption, is a common practice to delay or inhibit flowering of SDP in ornamental crop production [12, 13].

Similarly, low levels of constant light throughout the dark period, here called nighttime photons, can also disrupt flowering in SDP. P_fr_ plays a vital role in this process [14]; however, the mechanisms governing the response are only partially understood. It involves complex interactions between phytochromes (phyA, phyB and phyC in the SDP rice [15]) and 1) the circadian oscillator [16], 2) transcriptional regulation [17], and 3) possibly post-transcription stabilization [18].

Early studies that contributed to the discovery of phytochrome investigated the action spectrum of floral inhibition by night-break lighting in SDP. Using a spectrograph, these studies found strong inhibitory responses to red photons (600 to 700 nm), and minimal responses to the yet unnamed far-red photons, especially beyond 720 nm, although they still observed some inhibition at 770 nm; no response was observed at 840 nm [19]. Following the landmark flip-flop seed germination study with red and far-red photons [20], studies found that far-red could reverse red night-break inhibition of flowering in SDP [21, 22]. Although flowering was able to be re-induced by a far-red pulse after a red pulse, it differed from the germination response in that each additional cycle had a reduced response. After four cycles of alternating between red and far-red pulses, flowering was almost entirely inhibited [22]. Follow-up studies determined that high doses (dependent on intensity and duration) of far-red (with or without prior night-break with red) inhibited flowering of SDPs compared to a control without night-break lighting [22–25].

Table 1 summarizes the effect of far-red night-break lighting compared to controls without night breaks reported in studies spanning 63 years. The older studies report stage of floral development as an index, and the newer studies typically report time to flowering. Additionally, the older studies typically used a spectrograph with filters while the newer studies apply far-red photons with LEDs that have a peak at about 730 nm. It should be noted that this LED outputs some photons below 700 nm, while the NIR LED does not output photons below 700 nm. Some studies show a delay in flowering (see [26]), indicating that photons above 700 nm are able to activate phytochrome into P_fr_ and inhibit flowering. By contrast, some studies under similar conditions show no significant response (see [27]). These contradictions may be due to differences in the duration of the dark period, intensity of the far-red, duration of the night-break and sensitivity of the species (Table 1). Floral initiation is a complex molecular process, and different species/cultivars will have different thresholds for a photo-molecular process to occur. Therefore, it is important to choose species known to be sensitive to night-break/nighttime photons when investigating the ability of photons from NIR LEDs to activate phytochromes and inhibit flowering. Vince-Prue [11] listed soybean (*Glycine max*) and *Cannabis sativa* as among the most photosensitive species to nighttime photons.

**Table 1:** Summary of the effect of far-red night-break lighting on flowering development or time to flowering.

Species	Reported FR intensity	FR source	Night break length	Photoperiod conditions (day/night)	Effect on flowering development	Citation	Comment
*Xanthium pensylvanicum* Wallr.	unclear	filtered sunlight with output near 735 nm	12 min	12h/12h	Reduced stage of flowering from 6 to 4 (scale from 0 to 7) - 33% reduction	Downs [22]	Provided after R (about 50 μmol m^-2^ s^-1^)
*Chrysanthemum morifolium* cv. Indianapolis Yellow		Filtered incandescent			Reduced stage of flowering from 4.1 to 0.4 (scale from 0 to 10) - 90% reduction		
*Chrysanthemum morifolium* cv. Shasta	unclear		81 min	9h/15h	Reduced stage of flowering from 3.7 to 0 (scale from 0 to 10)—inhibited	Cathey and Borthwick [23]	
*Chrysanthemum morifolium* cv. Honey Sweet					Reduced stage of flowering from 3.7 to 0 (scale from 0 to 10)—inhibited		
*Chenopodium rubrum*	14 μmol m^-2^ s^-1^	Spectrograph centered at 730 nm (about 720 to 740 nm)	16 min	8h/16h	Reduced stage of flowering from 9 to 6.7 (scale from 0 to 9) - 26% reduction	Kasperbauer et al. [24]	
	about 50 μmol m^-2^ s^-1^	Filtered incandescent quantified from 710 to 800 nm		12h/12h	Reduced stage of flowering from 6.7 to 4.9 (scale from 0 to 7) - 27% reduction		
*Xanthium pensylvanicum* Wallr.			1 h	8h/16h	Reduced stage of flowering from 6.8 to 3.1 (scale from 0 to7) - 55% reduction	Mancinelli and Downs [25]	
				4h/20h	Reduced stage of flowering from 6.7 to 0.9 (scale from 0 to 7) - 87% reduction		
*Oryza sativa* (rice)	18000 μmol m^-2^	acrylic filtered fluorescent. Shortest wavelength ≈ 710 nm, peak ≈ 765 nm	"flash"	10h/14h	no effect	Ishikawa et al. [27]	
*Tagetes erecta* (African Marigold) cv. America Antigua Yellow	1.3–1.6 μmol m^-2^ s^-1^	LED peak at about 730 nm, quantified from 700 to 800 nm	4 h	9h/15h	9 day delay	Craig and Runkle [12]	only significant in one of two replicate studies
*Chrysanthemum morifolium* Ramat. cv. Reagan	62.5 μmol m^-2^ s^-1^	LED peak at about 740 nm, quantified from 300 to 900 nm	4 h	12h/12h	1.7 day delay	Higuchi et al. [28]	
*Chrysanthemum* ×*morifolium* cv. Adiva Purple	1.3–1.6 μmol m^-2^ s^-1^	LED peak at about 735 nm, quantified from 700 to 800 nm	4 h	9h/15h	no effect	Craig and Runkle [13]	Data for Dahlia should be interpreted with caution because there was incomplete flowering in SD and FR NB treatments
*Dahlia hortensis* cv. Carolina Burgundy					11 day delay		
*Dahlia hortensis* cv. Figaro Mix					8 day delay		
*Tagetes erecta* (African Marigold) cv. America Antigua Yellow					10 day delay		
*Chrysanthemum seticuspe*	20 μmol m^-2^ s^-1^	LED peak at about 740 nm	10 min	8h/16h	no effect	Higuchi et al. [29]	Supplementary data
*Chrysanthemum morifolium* Ramat. cv. Iwa no hakusen	6.6 μmol m^-2^ s^-1^	LED peak at 728 nm, quantified from 400 to 800 nm	6 h	12h/12h	Reduced stage of flowering from 0.86 to 0.27 (scale from 0 to 1) - 96% reduction	Liao et al. [30]	R NB similar to SD
*Chrysanthemum morifolium* Ramat. cv. Jimba					no effect		
*Dendranthema grandiforum* cv. Gaya Yellow	10 μmol m^-2^ s^-1^	LED peak at 730 nm	4 h	10h/14h	no effect	Park and Jeong [26]	

Results differed between studies, possibly due to the difference in treatments (also described). Stage of flowering refers to a flowering development index, different publications use different scales. R: Red; FR: Far-red; NB: Night break; SD: Short-day

Photons from NIR LEDs could also potentially affect plant growth and development by inactivating P_fr_ back into P_r_. Far-red photons are often reported to increase stem elongation [31], a process that is modulated through the inactivation of phytochrome [32].

We investigate the ability of photons with wavelengths greater than 700 nm from NIR LEDs applied over a 24 h photoperiod to 1) delay flowering in two sensitive short-day species, and 2) elongate stems. We found that at high enough doses, photons from NIR LEDs can affect both of these plant responses, indicating a role of long wavelength photons in modulating plant growth and development.

## Material and methods

### Plant materials

Soybean (*Glycine max* cv. Hoyt) were seeded into 1.7 L pots inside a greenhouse. Rooted cuttings of medicinal hemp (*Cannabis sativa* L. cv. T1 “Trump”) were transplanted into 6.5 L pots filled with a 3:1 mixture of peat/vermiculite. The media was amended with 1.6 g per L of dolomitic lime to bring the pH to 5.8 and 0.8 g per L Gypsum (CaSO_4_) to provide additional sulfur. Soybeans emerged four days after planting and were moved from the greenhouse into the growth chamber (CMP 3023, Conviron, Winnipeg, Canada). After transplanting, the *Cannabis* was grown in the greenhouse for one week (28/25 ˚C day/night; 18/6 h day/night) before moving into the growth chamber.

### Spectral treatments

A growth chamber (0.77 ×1.8 m) was split in half with white reflective cardboard to minimize light contamination between sections. The background spectrum for both sides was provided by white + red LEDs (Icarus Vi, BIOS, Melbourne FL), which had 10% blue (400 to 500 nm), 22% green (500 to 600 nm), and 68% red (600 to 700 nm). Two NIR LED fixtures (Ray 22 custom spectra; Fluence Bioengineering, Austin, TX) with a peak at about 850 nm were added to one side of the chamber. The other side received no NIR photons.

For soybean, two studies were conducted in time, one with a low NIR treatment [nighttime NIR photon flux density (700 to 900 nm) = 44 μmol m^-2^ s^-1^] and one with no added NIR, and a second study with a high NIR treatment (nighttime NIR photon flux density = 87 μmol m^-2^ s^-1^) and no added NIR treatment. Each study contained 12 plants per treatment. The *Cannabis* study was conducted across three studies in time. In addition to treatments with no added photons from the NIR LEDs, the first study contained a high night far-red flux density (nighttime NIR photon flux density = 62 μmol m^-2^ s^-1^) with four replicate plants and the second and third studies contained a low night far-red flux density (nighttime NIR photon flux density = 121 μmol m^-2^ s^-1^) with three replicate plants. All studies were conducted in the same split chamber with two treatments occurring consecutively.

The white + red background light was applied for a 12 h photoperiod and the NIR was applied for the full 24 h. NIR treatments began as soon as plants were moved into the growth chambers, and continued until the termination of the study. Measurements were made with a spectroradiometer (PS-300; Apogee instruments; Logan, UT) with 13 measurements made for each treatment. Spectral traces from the *Cannabis* study are shown in Fig 2. The spectral data is summarized in Table 2. To increase the accuracy of far-red measurements (700 to 800 nm) a high integration time was used to improve the signal to noise ratio of the spectroradiometer. Table 2 splits the flux of photons from NIR LEDs into three regions: FR-A (700 to 749 nm), FR-B (750 to 799 nm) and FR-C (800 to 900 nm). The treatment with no added NIR had some (an order of magnitude lower) flux of photons during the night period due to light leaking between the two halves of the chamber (Table 2). Nighttime PPE was calculated assuming only photoconversions (no thermal reversion; see more details in Discussion) using data from Kelly and Lagarias [6], Lagarias et al. [7] and Sager et al. [8]. Only data between 700 to 800 nm was used to calculate nighttime PPE. This is because 1) the SPD below 700 nm departed from log-linearity (LEDs have a Gaussian distribution meaning it should be log-linear), and 2) the flux of photons from the NIR LED below 700 nm is less than what would generally be present in moonlight (see Discussion and S1 Fig).

**Table 2:** Spectral analysis of NIR treatments.

	Soybean					
	---------------Day--------------			---------------Night--------------		
	no NIR	low NIR	high NIR	no NIR	low NIR	high NIR
PPFD (400–700 nm)	646	638	651	-	-	-
FR photon flux density						
FR-A (700–749 nm)	10	10	10	0.0	0.1	0.2
FR-B (750–799 nm)	3.2	4.8	7	0.1	1.7	3.8
FR-C (800–900 nm)	4.7	41	83	2.0	42	83
PPE						
Kelly and Lagarias [6]	0.87	0.87	0.87	-	0.03	0.03
Lagarias et al. [7]	0.86	0.86	0.86	-	0.04	0.04
Sager et al. [8]	0.88	0.88	0.87	-	0.16	0.15
	*Cannabis*					
	---------------Day--------------			---------------Night--------------		
	no NIR	low NIR	high NIR	no NIR	low NIR	high NIR
PPFD (400–700 nm)	832	840	837	-	-	-
FR photon flux density						
FR-A (700–749 nm)	13	14	14	0.0	0.1	0.3
FR-B (750–799 nm)	4.3	6.4	8.9	0.1	2.6	5.2
FR-C (800–900 nm)	5.8	55	114	2.0	59	116
PPE						
Kelly and Lagarias [6]	0.87	0.86	0.87	-	0.03	0.03
Lagarias et al. [7]	0.86	0.86	0.86	-	0.04	0.04
Sager et al. [8]	0.88	0.87	0.87	-	0.16	0.15

Values in this table represent averages from 13 measurements in each chamber. Additionally, treatments with the same level of NIR are averaged together. PPE was calculated using data from Kelly and Lagarias [6], Lagarias et al. [7] and Sager et al. [8]. Only wavelengths between 700 to 800 nm were used to calculate night PPE.

### Environmental conditions

**Fig 3:**
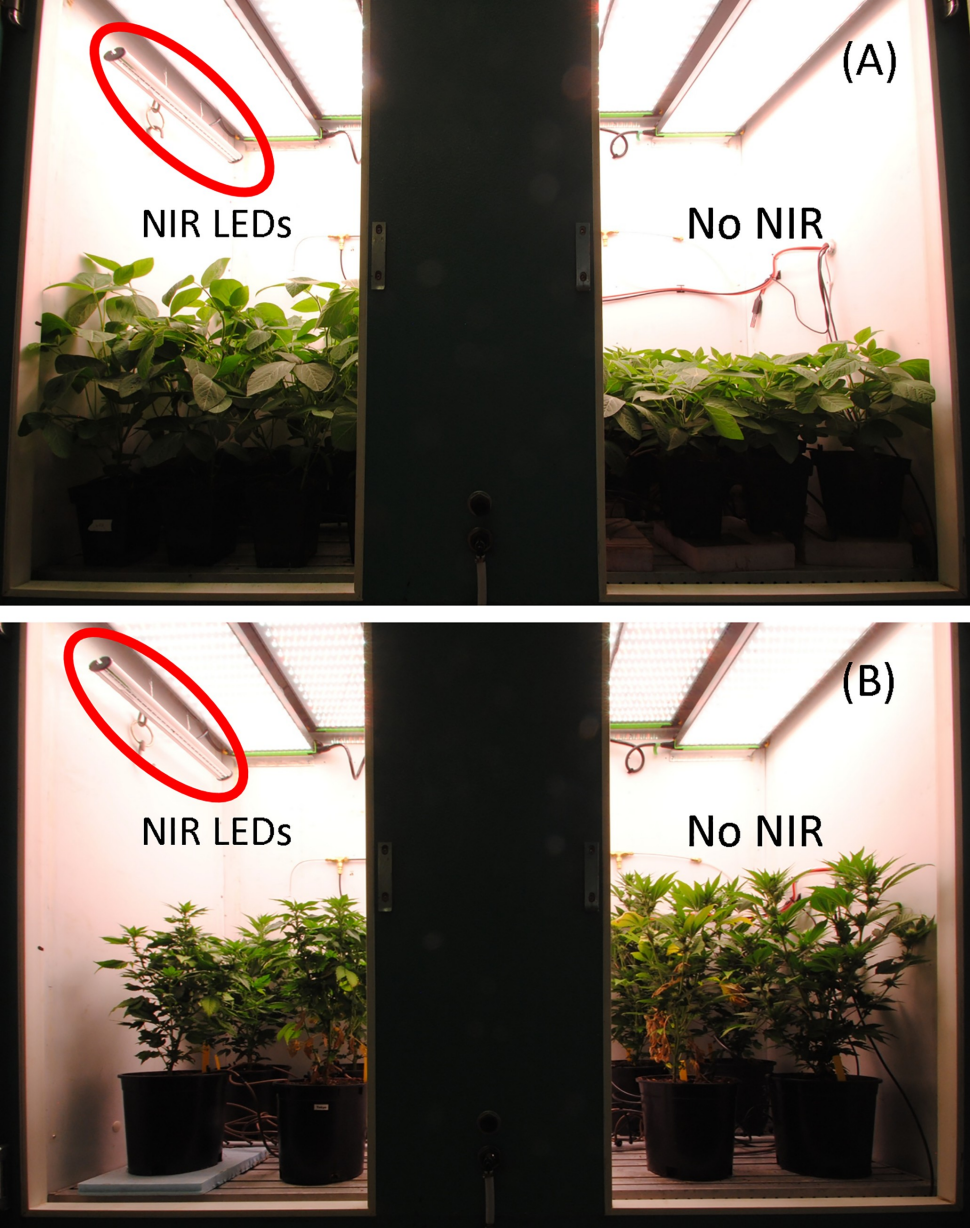
Photo at the end of the soybean (A) and *Cannabis* (B) study. The near infrared (NIR) LEDs, circled in red, were provided for the full 24 h, while the background light was provided for a 12 h photoperiod. The no NIR treatment had an order of magnitude lower flux of photons than the NIR treatments due to some light leaking from the NIR side of the chamber to the no NIR side (see Table 2).

Temperature was a constant 26°C day/night in the growth chambers (Fig 3). CO_2_ was maintained at 400 ppm. Inductive photoperiods (12/12 h day/night) began when plants were moved into the growth chambers. Plants were irrigated daily to a 10% excess with a complete liquid fertilizer [Peter’s Peat-lite professional 20-10-20 (20N-4.4P-16.6K), Everris NA, Inc., Dublin, OH] at a rate of 120 mg N per L. Greencare micronutrients (Greencare Fertilizers, Inc., Kankakee, IL) were added at a rate of 7 mg per L. AgSil 16H (PQ Corporation, Malvern, PA) was added using a second proportioner for the liquid fertilizer at a rate of 8.4 mg Si (0.3 mmol Si) per L. Electrical conductivity (EC) of the nutrient solution was 1.2 mS cm^-1^ and pH was 6.8.

### Plant measurements

Plants were monitored daily to determine time to flowering. In soybean time to flowering was defined by emergence of the first colored flower. In *Cannabis* time to flowering was defined as when the apical inflorescence reached 2 mm. Stem length of soybean was measured from the base of the stem to the apical meristem when flowering first occurred.

### Statistics

All data were analyzed using SigmaPlot graphical/statistical software (Systat Software, Inc., San Jose CA). All plants within each treatment were averaged together in each study and analyzed using linear regression. Linear regression was used because the treatment (photon flux density from the NIR LED) was a quantitative variable, not qualitative or categorical.

## Results and discussion

### Time to flower

**Fig 4:**
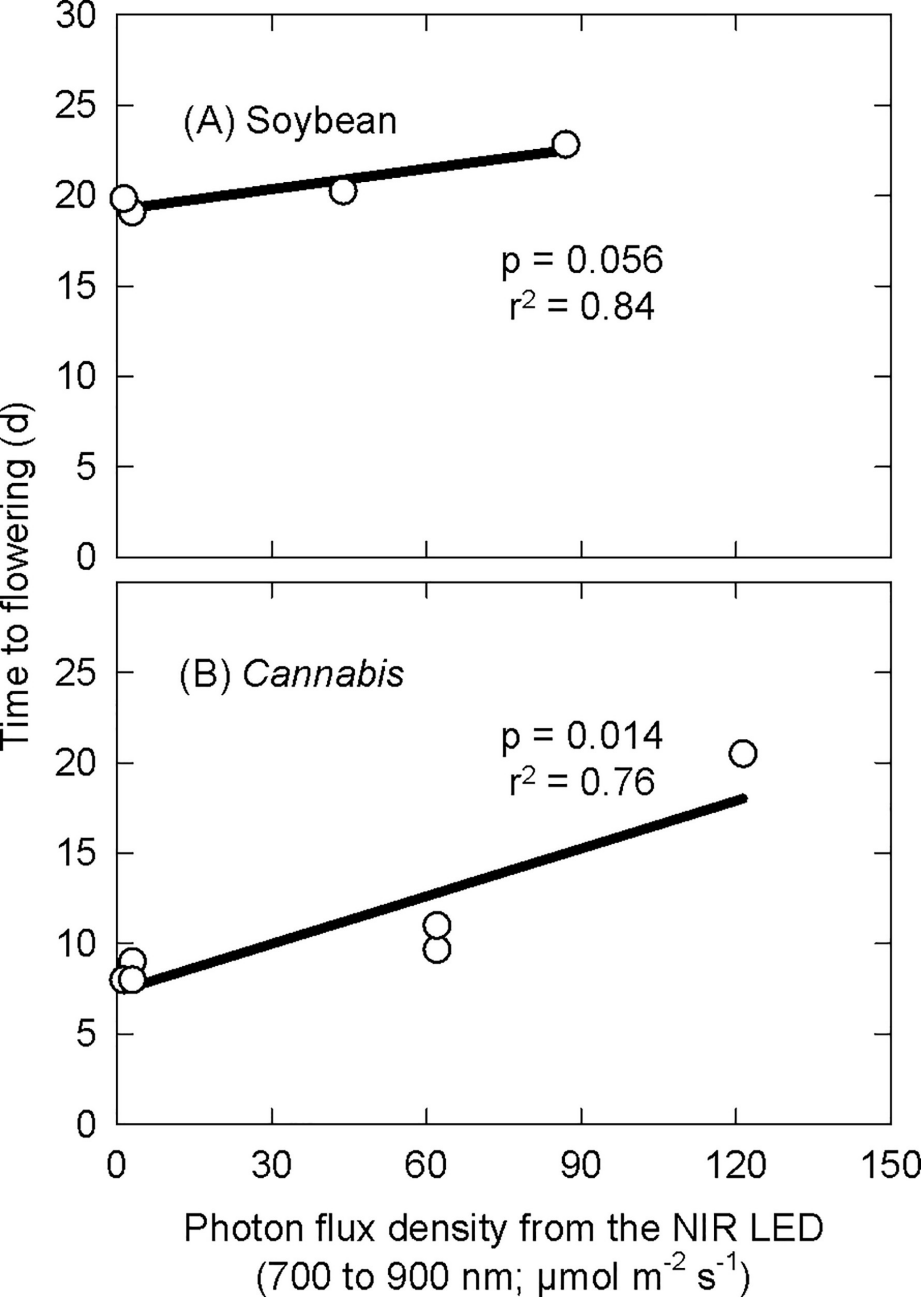
Effect of photons (700 and 900 nm) on time to flowering in (A) soybean and (B) *Cannabis*. Data points are the average effect within each treatment.

Increasing the photon flux density from NIR LEDs delayed flowering (increased time to flowering) in both soybean (p = 0.056) and *Cannabis* (Fig 4, p = 0.014). On average, the high NIR treatment delayed flowering of soybean and *Cannabis* by 3 and 12 d, respectively, compared to the lowest, no added NIR treatment. All soybean plants within each treatment flowered within three days of each other and all *Cannabis* plants within each treatment flowered within four days of each other. Plants were not rotated in the chambers, and thus only the average effect within the chamber was used for statistical analysis.

Previous studies have provided conflicting evidence regarding the effects of night-break photons beyond 700 nm on time to flower (Table 1). Flowering is a complex process, and the molecular/genetic mechanisms regulating photoperiodic flowering continue to be investigated. Many details of this process, as well as the universality of metabolic pathways remain uncertain [17]. Nevertheless, it is well established that phytochromes play an essential role in flowering [4], but these photoreceptor proteins act on at least three separate metabolic pathways: the circadian oscillator [16, 33], transcriptional regulation [17] and post-transcriptional stabilization [18]. Circadian control and transcription both require the nuclear localization of phytochrome. Only the P_fr_ form of phytochrome can enter the nucleus to disrupt flowering in conditions with night-break or nighttime photons. The necessary thresholds of P_fr_ to affect these responses are not known and likely differ among species [34]. Kasperbauer et al. [24] speculated from their data that just 1 to 2% of phytochrome in the active form for 60 minutes was enough to inhibit flowering in *Chenopodium rubrum*. Although the estimates of PPE using photoconversion cross-sections from Kelly and Lagarias [6], Lagarias et al. [7] and Sager et al. [8] were reasonable uniform for a single treatment during the day, they varied significantly for the night period (Table 2). This is largely due to the variability in the photoconversion cross-sections for P_r_ to P_fr_ between studies, especially Sager et al. [8] compared to the other two studies [6, 7]. Ignoring data from Sager et al. [8] due to its apparent inaccuracies above 750 nm (compare Fig 6 in [6] with Fig 5 in [8]), the photoconversion cross-sections and the SPD between 700 to 800 nm estimate that about 3–4% of the total pool of phytochrome was in the P_fr_ form during the night in this study (Table 2). These estimations of P_fr_ as a fraction of P_total_ are likely too high because 1) they likely contain inaccuracies at the longer wavelengths (above 750 nm) [3, 4], and 2) they do not include thermal reversion of P_fr_ back to P_r_.

Reversion/relaxation of P_fr_ back into P_r_ occurs in a non-photochemical process that is temperature dependent. This process was historically called dark reversion, but is now called thermal reversion. Thermal reversion has been well studied [35], but it has only recently been incorporated into estimates of P_fr_ to P_total_, especially in low light [36–38].

Jung et al. [39] determined that *Arabidopsis thaliana* phyB-P_fr_ had a half-life of about 52 minutes at 27 ˚C, the approximate temperature of this study. This half-life likely only applies to phyB at 27 ˚C. Warmer temperatures result in shorter half-lives compared to cooler temperatures. Additionally, different types of phytochromes have different stabilities. For example, phyA demonstrates thermal reversion in multiple species [35], phyD is thermally unstable, and phyE is highly thermostable [40]. Osugi et al. [10] determined that all phytochromes in rice (phyA, phyB and phyC) play a role in flowering, making it difficult to estimate the thermal reversion of the phytochromes in the species used in this study. Altogether, it was difficult to predict the nighttime PPE due to variation (and possible inaccuracy) in the photoconversion cross-sections, unknown thermal reversion rates, and spectral distortion within leaves [2, 24].

Nonetheless, the photoconversion cross-sections determined *in vitro* are not zero beyond 700 nm (Fig 1), indicating that some amount of P_r_ will be converted into P_fr_ during the night period with an application of NIR photons. The response of delayed flowering in two photosensitive species with the application of photons from an NIR LED is similar to classic very low fluence responses (VLFR), which require such low concentrations of P_fr_ (phyA) that they are both irreversible and able to be induced by far-red [9, 41]. Although some VLFRs can be induced by doses as low as 0.001 nmol m^-2^ [41], the intensity of full moonlight has been reported to range from 2 to 5 nmol m^-2^ s^-1^ [42, 43]. It would generally be disadvantageous for a SDP to be sensitive to moonlight, although there are exceptions [44]. Therefore we used a 1 nmol m^-2^ s^-1^ threshold below which photons from the NIR LED were considered ineffective for the response. Only photons above 700 nm were applied at high enough doses to cross this threshold. Separate from the possibility that the response can be explained as a VLFR, the effect could be categorized as a far-red induced high irradiance response (FR-HIR), which are defined as responses that are proportional to the photon flux density and show a peak responsivity in the far-red region [41]. Investigation into the FR-HIR response has shown that it requires cycling of phyA from P_r_ to P_fr_ back to P_r_ [45]. This is because phyA is shuttled into the nucleus by the proteins FHY1 and FHL. Only the P_fr_ form of phyA interacts with FHY1/FHL, thus conversion of P_r_ to P_fr_ enables transportation of phyA into the nucleus, after which P_fr_ to P_r_ conversion disassociates phyA from FHY1/FHL, allowing phyA to accumulate in the nucleus [46]. To be active in the nucleus, phyA likely requires further activation from P_r_ to P_fr_ [32, 46]. It is noteworthy that this response still requires the activation of phytochrome from P_r_ to P_fr_, which could have been driven by photons from the NIR LED. Neither the VLFR nor the FR-HIR responses have been assessed in the species investigated here, especially in the context of phytochrome mutants, thus the exact mechanism of action remains to be determined.

An additional consideration is that it is possible that applying the photons from the NIR LEDs only during the dark period (instead of both the light and dark period for 24 h) could have potentially resulted in a different response. But, the delay in flowering by photons from the NIR LEDs was most likely caused by the activation of phytochrome during the dark period.

There are concerns in the *Cannabis* industry that photons from NIR LEDs cause monecious flowering. *Cannabis* is naturally dioecious; only female plants are desired for medical *Cannabis* cultivation. Monoecious flowering is often confused with hermaphroditism. Botanically, these terms are distinct: monoecious refers to the presence of separate male and female flowers on the same plant, while hermaphrodite refers to the presence of both male and female reproductive organs within an individual flower [47]. In practice, the distinction is not important because both monoecious and hermaphroditic *Cannabis* produce pollen and potentially reduce product quality and value [48]. The tendency of *Cannabis* to form monoecious or hermaphroditic plants is under genetic and environmental influence [49, 50]. No monoecious or hermaphrodite plants were observed in this study, but we did not grow the plants to maturity.

### Stem length

**Fig 5:**
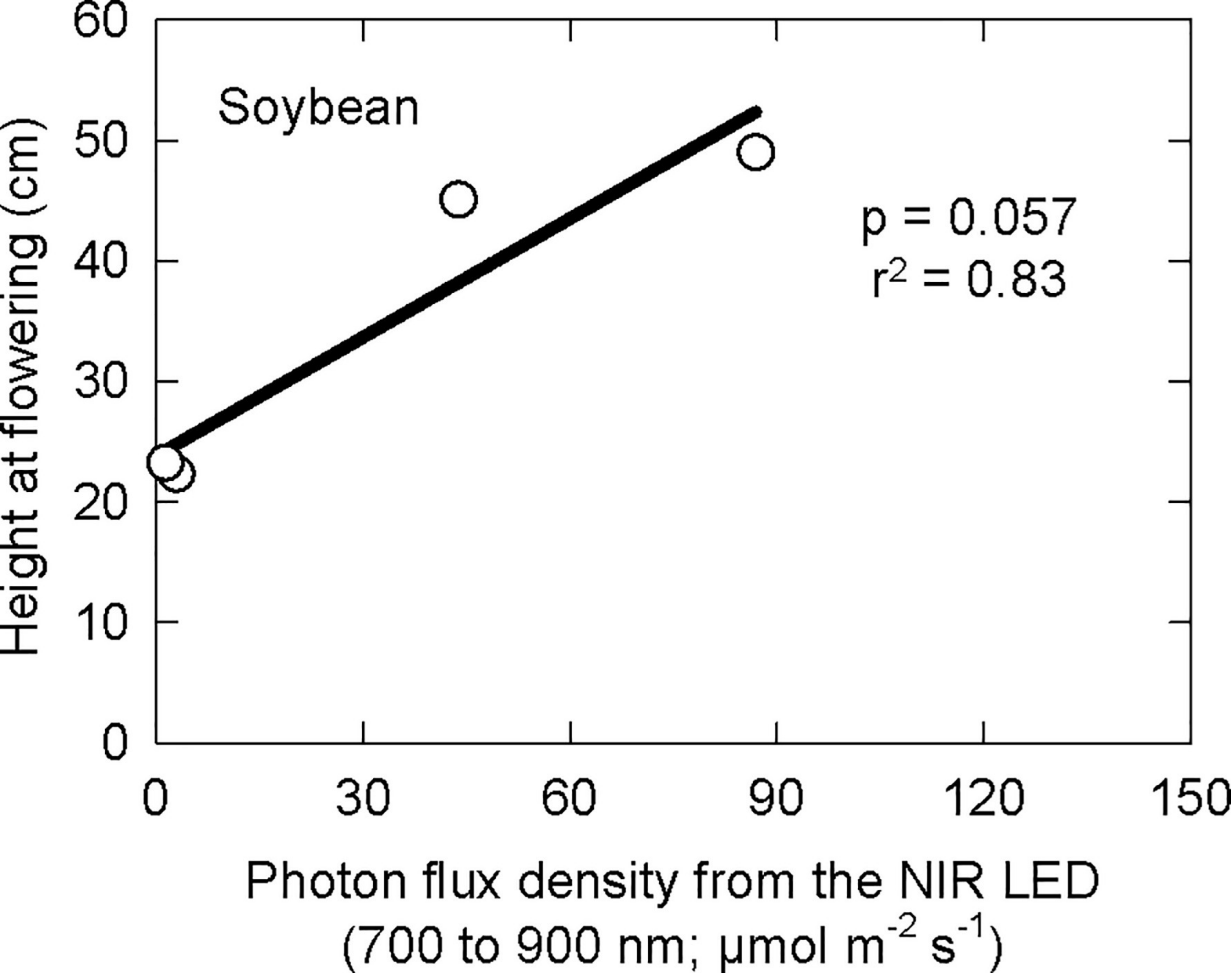
Effect of photons (between 700 and 900 nm) on soybean height at flowering. Data points are the average effect within each treatment.

Soybean plant height at flowering was increased by photons from NIR LEDs (Fig 5). The coefficient of variation (standard deviation divided by the mean) of plant height at flowering in each treatment was at most 0.15.

Far-red photons have a significant effect on stem elongation in soybeans [31], so the effect of photons from NIR LEDs on soybean plant height (p = 0.057) was not surprising (Figs 3 and 5). P_fr_ inhibits the activity of transcription factors involved in stem elongation meaning that this elongation response is caused by the inactivation of phytochrome, P_fr_ to P_r_ [32]. It is important to note that inhibition of stem elongation and inhibition of flowering require different thresholds of P_fr_.

**Fig 6:**
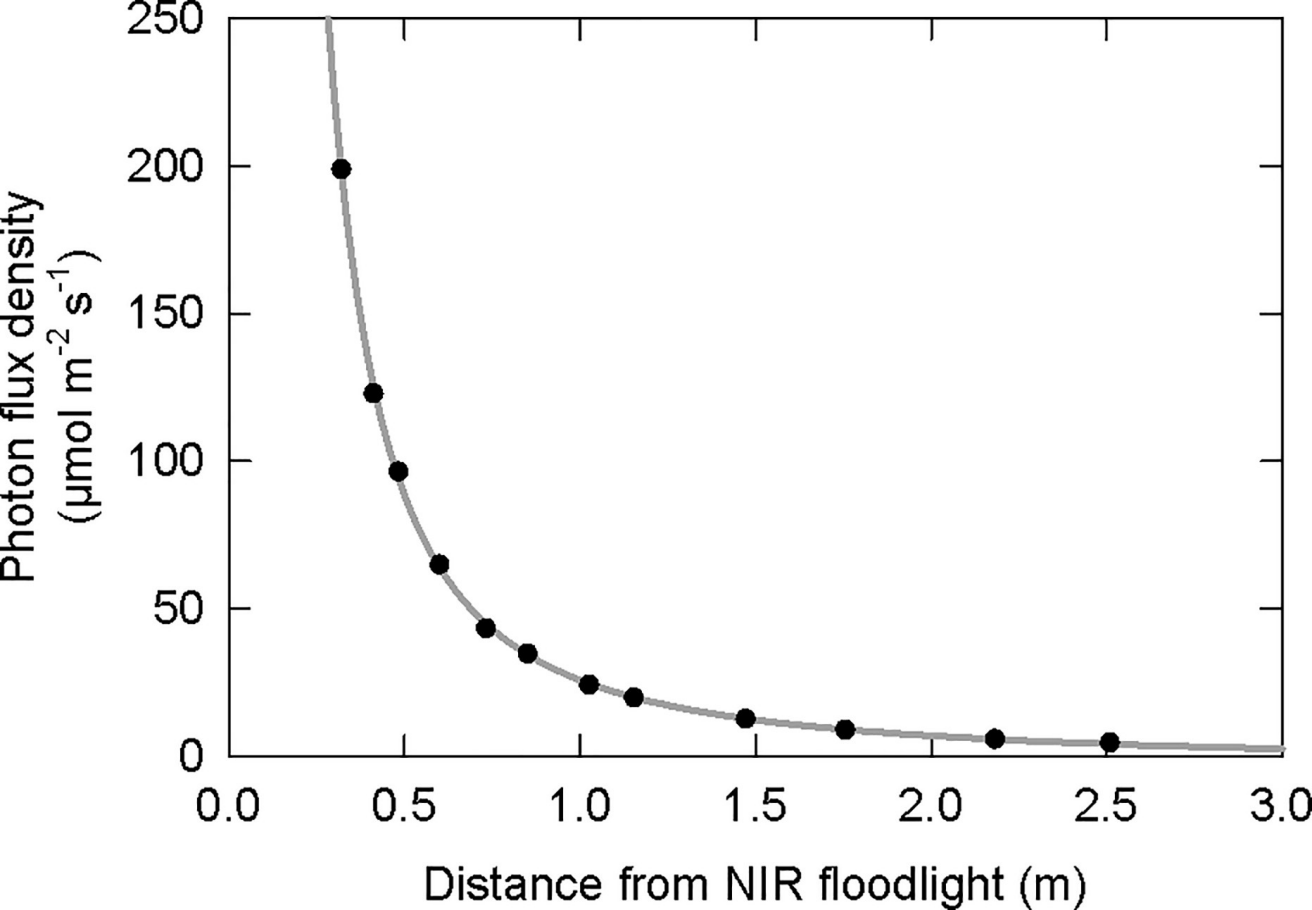
Total photon flux density from an NIR LED floodlight as a function of distance. These measurements were made directly below the floodlight at increasing distances, and they follow the inverse square law.

We conclude that photons from NIR LEDs applied for 24 h per day can both inactivate P_fr_ to P_r_ inducing stem elongation and activate P_r_ to P_fr_ delaying flowering in sensitive SDP. For practical applications, this means that the NIR LEDs in security cameras for night vision in controlled environment agriculture have the potential to alter plant development. We measured the photon flux from an NIR floodlight, which is used to increase the range of night vision for a security camera, to determine the intensities that plants might be exposed to in commercial setting (Fig 6). The total photon flux density at one meter from the floodlight was about 25 μmol m^-2^ s^-1^. Our data indicate that this intensity may be enough to delay flowering by one day in soybean and two days in *Cannabis*. Additionally, this intensity from the NIR LEDs is enough to increase stem elongation by 33% in soybean. It should be noted that these measurements were made with a floodlight, which represents a much higher flux of photons compared to the photon flux of a security camera–although, floodlights can be used in controlled environment settings. Additionally, most plants would not be within one meter of the NIR LEDs. By a distance of about 3 m, the photon flux from these LEDs drops to about one μmol m^-2^ s^-1^, which is likely too low to have any noticeable effects. Therefore, although NIR photons from security cameras have the potential to affect plant growth and development, intensities are likely too low to have an effect in most practical settings–especially on less photosensitive species.

## Supporting information

S1 Fig Spectral photon distribution (SPD) from the highest near infrared (NIR) intensity used across all the treatments. The spectrum is plotted on a log scale. Because LEDs output a Gaussian distribution, the tail of the LED ought to be a straight line on a log scale. This indicates that as the measured SPD changes from linear (715 to 800 nm) to non-linear (550 o 715 nm), the data is primarily caused by either a) stray light in the spectroradiometer, and/or b) instrument noise. We model what the spectrum ought to be with a dashed red line. The photon flux density of full moonlight has been reported to be between 2 and 5 nmol m^-2^ s^-1^ [42, 43], and Kadman-Zahavi and Peiper [44] reported that moonlight was able to affect flowering in highly sensitive SDP. Thus, it seems useful to use an intensity lower than full moonlight as a threshold below which photons are unlikely to have an effect. Additionally, although some very low fluence responses are sensitive to intensities lower than moonlight, it seems evolutionarily disadvantageous to be sensitive to these intensities for flowering responses. We use 1 nmol m^-2^ s^-1^ nm^-1^ as the intensity threshold below which flowering is assumed not affected. With this consideration, 700 nm was the cutoff wavelength. Integrating the modeled spectral output (dashed red line) between 650 and 700 nm does provide a photon flux density of about 10 nmol m^-2^ s^-1^. This could theoretically induce a response, but we assume they do not.
(PDF)

S1 Data (XLSX)
