# Supplementary material for: Photons from NIR LEDs can delay flowering in short-day soybean and Cannabis: Implications for phytochrome activity
Source: PLoS One. 2021 Jul 27;16(7):e0255232. doi: 10.1371/journal.pone.0255232 (PMC8315492; doi:10.1371/journal.pone.0255232)
Supplement: S1 Fig — The spectrum is plotted on a log scale. Because LEDs output a Gaussian distribution, the tail of the LED ought to be a straight line on a log scale. This indicates that as the measured SPD changes from linear (715 to 800 nm) to non-linear (550 o 715 nm), the data is primarily caused by either a) stray light in the spectroradiometer, and/or b) instrument noise. We model what the spectrum ought to be with a dashed red line. The photon flux density of full moonlight has been reported to be between 2 and 5 nmol m-2 s-1 [42, 43], and Kadman-Zahavi and Peiper [44] reported that moonlight was able to affect flowering in highly sensitive SDP. Thus, it seems useful to use an intensity lower than full moonlight as a threshold below which photons are unlikely to have an effect. Additionally, although some very low fluence responses are sensitive to intensities lower than moonlight, it seems evolutionarily disadvantageous to be sensitive to these intensities for flowering responses. We use 1 nmol m-2 s-1 nm-1 as the intensity threshold below which flowering is assumed not affected. With this consideration, 700 nm was the cutoff wavelength. Integrating the modeled spectral output (dashed red line) between 650 and 700 nm does provide a photon flux density of about 10 nmol m-2 s-1. This could theoretically induce a response, but we assume they do not. (PDF) [file pone.0255232.s001.pdf]

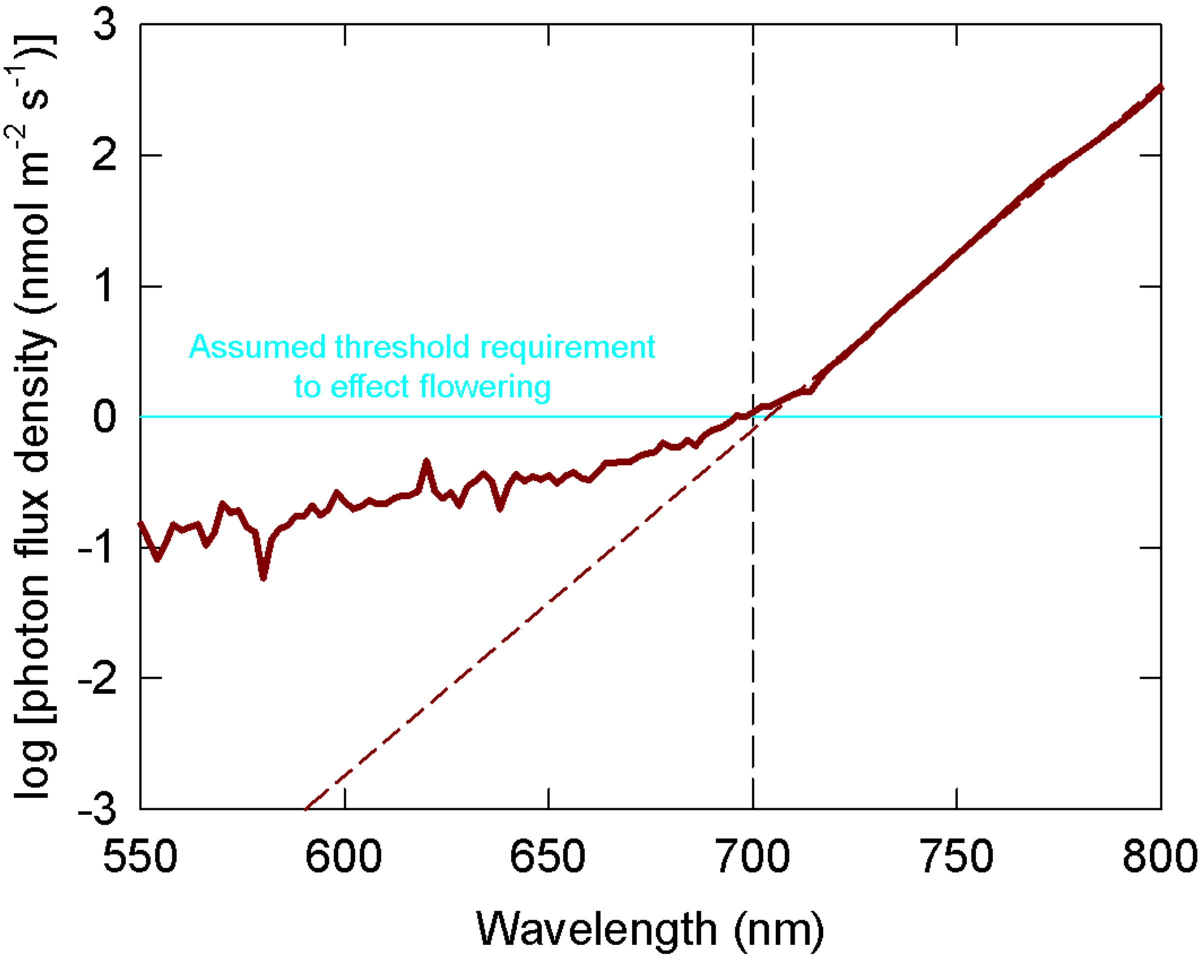

S1 Fig. Spectral photon distribution (SPD) from the highest near infrared (NIR) intensity used across all the treatments. The spectrum is plotted on a log scale. Because LEDs output a Gaussian distribution, the tail of the LED ought to be a straight line on a log scale. This indicates that as the measured SPD changes from linear (715 to 800 nm) to non-linear (550 o 715 nm), the data is primarily caused by either a) stray light in the spectroradiometer, and/or b) instrument noise. We model what the spectrum ought to be with a dashed red line. The photon flux density of full moonlight has been reported to be between 2 and 5 nmol m^{-2} s^{-1} [42,43], and Kadman-Zahavi and Peiper [44] reported that moonlight was able to affect flowering in highly sensitive SDP. Thus, it seems useful to use an intensity lower than full moonlight as a threshold below which photons are unlikely to have an effect. Additionally, although some very low fluence responses are sensitive to intensities lower than moonlight, it seems evolutionarily disadvantageous to be sensitive to these intensities for flowering responses. We use 1 nmol m^{-2} s^{-1} nm^{-1} as the intensity threshold below which flowering is assumed not affected. With this consideration, 700 nm was the cutoff wavelength. Integrating the modeled spectral output (dashed red line) between 650 and 700 nm does provide a photon flux density of about 10 nmol m^{-2} s^{-1}. This could theoretically induce a response, but we assume they do not.
